# Using Mobile Devices to Deliver Lifestyle Interventions Targeting At-Risk High School Students: Protocol for a Participatory Design Study

**DOI:** 10.2196/14588

**Published:** 2020-01-06

**Authors:** Ulrika Müssener, Marie Löf, Preben Bendtsen, Marcus Bendtsen

**Affiliations:** 1 Department of Medical and Health Sciences Linköping University Linköping Sweden; 2 Department of Biosciences and Nutrition Karolinska Institutet Huddinge Sweden

**Keywords:** mHealth intervention, lifestyle behavior, high school students, qualitative methods, participatory design

## Abstract

**Background:**

Unhealthy lifestyle behaviors such as insufficient physical activity, unhealthy diet, smoking, and harmful use of alcohol tend to cluster (ie, individuals may be at risk from more than one lifestyle behavior that can be established in early childhood and adolescence and track into adulthood). Previous research has underlined the potential of lifestyle interventions delivered via mobile phones. However, there is a need for deepened knowledge on how to design mobile health (mHealth) interventions taking end user views into consideration in order to optimize the overall usability of such interventions. Adolescents are early adopters of technology and frequent users of mobile phones, yet research on interventions that use mobile devices to deliver multiple lifestyle behavior changes targeting at-risk high school students is lacking.

**Objective:**

This protocol describes a participatory design study with the aim of developing an mHealth lifestyle behavior intervention to promote healthy lifestyles among high school students.

**Methods:**

Through an iterative process using participatory design, user requirements are investigated in terms of technical features and content. The procedures around the design and development of the intervention, including heuristic evaluations, focus group interviews, and usability tests, are described.

**Results:**

Recruitment started in May 2019. Data collection, analysis, and scientific reporting from heuristic evaluations and usability tests are expected to be completed in November 2019. Focus group interviews were being undertaken with high school students from October through December, and full results are expected to be published in Spring 2020. A planned clinical trial will commence in Summer 2020. The study was funded by a grant from the Swedish Research Council for Health, Working Life, and Welfare.

**Conclusions:**

The study is expected to add knowledge on how to design an mHealth intervention taking end users’ views into consideration in order to develop a novel, evidence-based, low-cost, and scalable intervention that high school students want to use in order to achieve a healthier lifestyle.

**International Registered Report Identifier (IRRID):**

DERR1-10.2196/14588

## Introduction

### Health Habits Among Youths and the Need for Scalable Interventions

The lifestyles of young people affect not only their current health but also their risk of a number of noncommunicable diseases (NCDs) such as cardiovascular diseases, cancers, chronic respiratory diseases, and diabetes. Insufficient physical activity, unhealthy diet, smoking, and harmful use of alcohol are all modifiable behaviors that increase the risk of NCDs [1-3]. Swedish national surveys have revealed that the majority of young people in Sweden do not consume the recommended daily amount of fruits and vegetables nor do they meet the recommended physical activity guidelines [4-6]. Also, smoking remains a global public health issue, and there is a high prevalence of smoking in youth [7] that also applies to Sweden [6]. Alcohol consumption has declined, but heavy episodic drinking continues to be a problem among alcohol-drinking adolescents [4,8]. Clearly, effective and evidence-based interventions to promote healthier lifestyles in adolescents are warranted.

Adolescence is characterized by rapid physical and psychological changes, together with increasing demands and influences of peers, school, and wider society. It is well documented that behaviors developed during this period influence health in adulthood. Adolescence is the peak period for initiation of substance use, which creates large health burdens in this age group [9,10]. As unhealthy lifestyle behaviors tend to be established in early childhood and adolescence and track into adulthood [11-13], efforts for outreach to high school students are vital. The prevention of diseases related to modifiable behavior has been emphasized as a key component of adolescent health [14]. Part of the success to reduce NCDs requires helping individuals to change their lifestyles to promote health [1,3,10]. According to the World Health Organization (WHO), the education sector can play an important role in health promotion for youths [15]. School health systems, with qualified professionals such as school nurses, welfare officers, and health educators, provide services for students that promote optimum health for their academic success. School multidisciplinary teams provide good accessibility for adolescents and a natural setting for attempting to endorse healthy lifestyle behaviors for as many adolescents as possible [16]. However, to be delivered by school health professionals, interventions require minimal resources and time.

### Mobile Health Interventions to Promote a Healthier Lifestyle Among Youths

Over the past decade, interest has increased in providing lifestyle interventions via mobile phones, often referred to as mobile health (mHealth) interventions. mHealth is defined by WHO as a medical or public health practice that is supported by mobile devices [15]. Major advantages with mHealth interventions are that they require fewer resources than traditional face-to-face interventions and they can be delivered at any time. To date, most mHealth interventions have focused on improving one or two lifestyle behaviors such as nutrition and/or physical activity or smoking cessation. However, there is also evidence that lifestyle behaviors may cluster (ie, individuals may be at risk from more than one lifestyle behavior) [17-19]. Interventions targeting multiple lifestyle behaviors at the same time may be beneficial for improving general lifestyle among adults [19,20] and may be more effective and efficient than those targeting a single behavior [21].

To date, although young people are early adopters of technology and frequent users of mobile phones, studies on interventions that use mobile devices to deliver multiple lifestyle interventions to high school students are few, and most interventions target only one or two single behaviors [22]. In this context, it is also relevant to note that a meta-analysis [23] examined the effectiveness of text message–based interventions for tobacco and alcohol cessation within a young adult population. Only 5 of the 14 studies reported significant differences between groups of substance use behavior outcomes. The authors concluded that the included randomized controlled trials (RCTs) lacked detail regarding intervention content. Consequently, replication of the RCTs and the possibility of identifying why and how previous interventions in youth were effective are difficult.

### Formative Research Processes

A neglected area of research is the documentation and critical analysis of the formative research processes required in the development and refinement of effective mHealth interventions [24]. A systematic review stressed the need for further research to evaluate the efficacy and effectiveness of intervention approaches in promoting preventive behavior among adolescents [25]. A more recent systematic review emphasized the urgent need to examine development processes for mHealth interventions. The authors concluded that it is important to fully understand how interventions have been developed to allow replication and adaptation of interventions across settings [26].

The prompt expansion of device capability presents many challenges for developers of mHealth interventions, especially when designing interventions that aim to affect multiple individual lifestyle behaviors [27]. As described in Bock et al [27], one set of challenges concerns the structure, content, and tone of the intervention. Previous research has pointed out that the most important factors during the design process are to be flexible and responsive to the input and feedback of the target audience: if they do not enjoy the program they may disengage [28]. A systematic review called for greater transparency in use of theory in developing mHealth interventions [29]. An additional challenge is that of technological cultural consistency (ie, to ensure the developed interventions and modes of access are compatible with the ways in which the intended target group uses technology) [27]. Given the identified challenges and needs regarding development of mHealth interventions, research is needed on how best to design mHealth interventions taking end user assessments into account [28].

### Aim

The aim of this research protocol is to describe the research process in developing a novel mHealth intervention to change risky lifestyle behaviors among high school students (LIFE4YOUth).

LIFE4YOUth is one of seven mHealth interventions in a research program (funded by Forte 2018-01410; principal investigator: ML) aiming to promote healthy eating, physical activity, smoking cessation, and nonrisky drinking in seven different populations in the health care system [30]. All included studies will follow a harmonized procedure for intervention development, and hence a secondary aim of this protocol is to describe the formative work for LIFE4YOUth as a framework for the included interventions in the research program.

## Methods

### Study Overview

**Figure 1:**
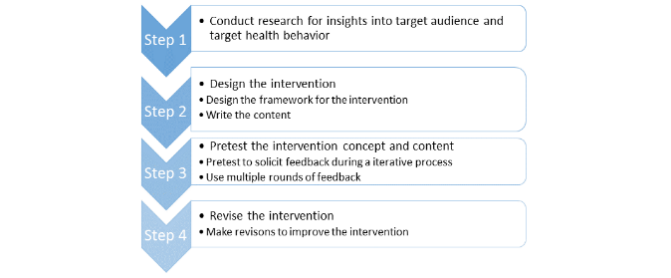
A simplified picture of the steps for developing an mHealth intervention (inspired by Abroms et al 2015).

The development of the LIFE4YOUth intervention is based on a review of the literature and will be inspired by the same phases of development and evaluation as any intervention provided by the National Institutes of Health [31] and further developed and described as recommended by Abroms et al [28] in their guide based on collective experiences in designing, developing, and evaluating mHealth interventions (Figure 1). The recommended steps for developing mHealth interventions include (1) conduct research for insight into target audience and target health behavior, (2) design the intervention, (3) pretest the intervention, and (4) revise the intervention [28]. This paper will focus on step 2 (designing the intervention) and step 3 (pretesting the intervention). The intervention will be designed and pretested during an iterative process, involving multiple rounds of feedback.

### Procedures

#### Preparations: Development of a Preliminary Version of LIFE4YOUth

The structure of a preliminary version of LIFE4YOUth was developed in early 2019. The intervention aims to target physical activity, diet, alcohol consumption, and smoking by giving high school students access to a mobile phone app to promote a healthy lifestyle. The structure and content are based on current best practices gathered from scientific literature on lifestyle interventions and behavior change and are inspired by fundamental theoretical constructs such as behavior change theories and psychological models [32,33]. The technical platform is based on our previous research in developing mHealth interventions [34-36].

#### Participatory Design Processes

**Figure 2:**
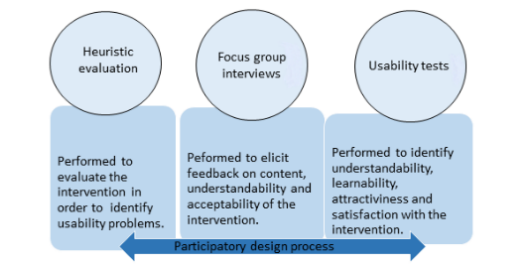
Activities in the development process of LIFE4YOUth.

The participatory design process used in this study will include three activities: (1) heuristic evaluation, (2) focus group interviews, and (3) usability tests. We will invite end users including high school students and university students and employees at Linköping University. The knowledge, experiences, ideas, and skills of the participants will be used to revise the intervention. Figure 2 presents the activities included in the design of the intervention.

#### Heuristic Evaluation

Effectiveness, as part of usability defined by the International Organization for Standardization standard 9421-11:2018, will be investigated using heuristic evaluation [37], a usability inspection method. During heuristic evaluation, trained evaluators review an intervention to find usability problems, assign them to a specific category of heuristics, and ascribe a severity rating in order to provide distinct usability information. The experts recruited for this evaluation are not necessarily usability experts but should have some level of expertise with the subject matter or technology required to use the investigated app. The heuristics will identify usability issues such as problems with unclear functions, confusing navigation, and consistency issues [38-40].

#### Focus Group Interviews

Initially, a series of focus groups will be conducted to enable the collection and analysis of three complementary forms of data: individual data, group-level data, and data generated from participant interactions [41,42]. Focus groups are semistructured discussions with research participants that aim to explore a specific set of issues [43]. The focus groups will be designed to elicit feedback on content, understandability, and acceptability of the proposed intervention so that modifications can be made.

#### Usability Tests

Usability tests [44-46] will be completed in order to further modify and improve the intervention. Usability tests consist of a human-computer interaction and refer to evaluating an intervention by testing it with potential end users with the goal of identifying understandability, learnability, and attractiveness and determining participant satisfaction with the intervention. The usability tests will provide information on whether participants are able to complete specified tasks successfully, identify how long it take to complete tasks, and identify changes required to improve user performance and satisfaction [44,47].

### Setting, Participants, and Recruitment

#### Heuristic Evaluation

Participants for the heuristic evaluation will be recruited by members of the research team through paper advertising (posters) in public areas at Linköping University. Participants will register their interest by contacting the research leader by email.

#### Focus Groups and Usability Tests

School staff at five high schools selected for convenience in Östergötland (Sweden) will be contacted via email and informed about the research project. Approximately 1000 students, both female and male, aged 15 to 18 years, attend these high schools and will all be invited to take part in the focus group interviews and usability tests. High school students at the selected schools are expected to be similar to the overall target population for mHealth interventions.

Participants among high school students will be recruited by the school staff through paper advertising (posters and leaflets), digital advertising (student email and school website), and information in the classrooms. High school students will register their interest by contacting the research leader by email or telephone or by contacting school staff who will send students the telephone number of the research leader.

#### Selection Criteria

Inclusion criteria for the heuristic evaluation will include university students and employees at the Faculty of Medicine and Health Sciences at Linköping University who are willing to participate and who own a mobile phone. Inclusion criteria for the focus group interviews and usability tests will include high school students aged 15 to 18 years at selected high schools in Östergötland who are willing to participate and who own a mobile phone. A total of 32 to 44 participants are expected. Exclusion criteria for the focus group interviews and usability tests will be high school students who are not Swedish-speaking or do not own a mobile phone.

### Data Collection

#### Heuristic Evaluations

A total of 15 heuristic evaluators, both students and employees at Linköping University, will be recruited. A research assistant will give participants a short (eg, 45 minutes) training session to instruct them on the main principles of heuristic evaluation during a meeting with all participants. The introduction will take place in a conference room at Linköping University. For the heuristic evaluation, a set of 10 standardized heuristics published by Nielsen [37] will be used. The heuristics for usability evaluation according to Nielsen are listed in Textbox 1.[37]

Participants will be taught how to use the heuristics to evaluate the intervention. All participants will be sent a link with a prototype of the intervention. Each evaluator will go through the prototype one time and independently identify issues tied to a specific heuristic (eg, visibility of system status, plain language, flexibility and efficiency of use, aesthetic design) and give them a severity rating [38-40]. The evaluation will be performed wherever the participants prefer and sent back to the research assistant in a prepaid envelope within 1 week of receipt. Heuristic evaluations will be gathered in May 2019.

**Heuristics for usability evaluation according to Nielsen.**

- Visibility of system status: system should always keep users informed about what is going on through appropriate feedback within reasonable time.
- Match between system and the real world: system should speak the users’ language with words, phrases, and concepts familiar to the user rather than system-oriented terms. Follow real-world conventions, making information appear in a natural and logical order.
- User control and freedom: users often choose system functions by mistake and will need a clearly marked “emergency exit” to leave the unwanted state without having to go through an extended dialog. Support undo and redo.
- Consistency and standards: users should not have to wonder whether different words, situations, or actions mean the same thing.
- Error prevention: even better than good error messaging is a careful design that prevents problems from occurring in the first place. Either eliminate error-prone conditions or check for them and present users with a confirmation option before they commit to the action.
- Recognition rather than recall: minimize users’ memory load by making objects, actions, and options visible. The user should not have to remember information from one part of the dialog to another. Instructions for use of the system should be visible or easily retrievable whenever appropriate.
- Flexibility and efficiency of use: accelerators, unseen by the novice user, may often speed up the interaction for the expert user such that the system can cater to both inexperienced and experienced users. Allow users to tailor frequent actions.
- Aesthetic and minimalist design: dialogs should not contain irrelevant or rarely needed information. Every extra unit of information in a dialog competes with relevant units of information and diminishes their relative visibility.
- Help users recognize, diagnose, and recover from errors: error messages should be expressed in plain language (no codes), precisely indicate the problem, and constructively suggest a solution.
- Help and documentation: even though it is better if the system can be used without documentation, it may be necessary to provide help and documentation. Any such information should be easy to search, be focused on the user’s task, list concrete steps to be completed, and not be too large.

#### Focus Group Interviews

A total of 4 focus group interviews with 3 to 6 participants in each group (12 to 24 participants) will be conducted between May and June 2019. Teachers will not be present during the interviews. The consolidated criteria for reporting qualitative research (COREQ) 32-item checklist [48] will be applied to give an explicit and comprehensive structure to the focus group interviews according to the following domains:

- Research team and reflexivity: a female researcher with a PhD degree and training and experience in qualitative methodology (UM) will be responsible for conducting the focus groups. A female observer (AS) will ask complementary questions at the end of the interview. The interviewer will explain the purpose of the interview and her interests in doing the research.
- Study design: an explorative qualitative approach [41,42] will be used for methodological orientation. All focus group interviews will be conducted in a high school setting. Each semistructured interview will be audiorecorded and will last approximately 1.5 hours. An interview guide (Multimedia Appendix 1) will be used [41]. Interview questions will be framed around the following domains: (1) making lifestyle changes, (2) use of the mobile phone for health informatics, (3) intervention content, (4) overall feedback, and (5) visual prototype. After discussing the first four domains, UM will present a low-fidelity paper-based prototype [49] including a series of printouts of the LIFE4YOUth program.
- Analysis and findings: analyses will be performed using systematic thematic analyses (further described under Data Analysis). Data will be coded by two researchers. Themes will not be identified in advance but will derive from the data. Quotations will be used to illustrate the themes and elucidate the findings.

#### Usability Tests

A total of 5 usability tests [45-47] will be completed. Five high school students will go through a 60-minute session during which all interactions with the intervention are videorecorded. A high-fidelity prototype [49], including the actual software start page, menu page, and 4 intervention modules (alcohol, smoking, physical activity, and diet) will be used. During this high-fidelity prototype testing, participants will go through the entire intervention module. A research assistant will ask participants to complete tasks while explaining their actions using a think aloud method [50,51]. An observer (AS) will note potential issues as the given tasks are performed by the participants. The assistant will not offer any help during the task execution to minimize any disruptions of spontaneous thoughts as well as to avoid bias in the results. After completing the session, the participants will be asked to complete a paper version of the system usability scale (SUS). The SUS is a standardized tool to get a global view of the participants’ subjective assessments of usability based on 10 questions [52]. The tests will be run on an iPhone and take place in June 2019 in a medical informatics lab room.

### Data Analyses

#### Heuristic Evaluation

All issues from evaluators will be pooled with potential duplicates merged and issues with high average severity ratings rectified [37,53]. Descriptive statistics will be used to summarize heuristic violations and associated severity scores.

#### Focus Group Interviews

Transcripts will be analyzed thematically in an iterative process of coding [54]. Analyses will focus on end user experiences and opinions regarding making lifestyle changes using the mobile device as a health tool, as well as on content, structure, and implementation of LIFE4YOUth. Systematic thematic analyses will follow a prescribed, sequential process: (1) noting overall impressions, (2) reducing and coding into themes, (3) searching for patterns and interconnections, (4) mapping and building themes, and (5) drawing conclusions. In order to ensure validity of the results and prevent bias in the qualitative analysis process, data will be independently coded by two researchers with a consensus reached by adjudication [41].

#### Usability Tests

After all user tests have been completed, observers and other members of the research group will discuss whether specific tasks stood out or hindered the progress in development of the program. Analysis of the videorecordings will be informed inspired by inductive program theory development [55].

Analysis will focus on features of the intervention related to design, format, instructions, navigation, terminology, and learnability that need to be redesigned. Descriptive statistics will be used to analyze problem counts and time taken. Average scores from the SUS will be used to identify average satisfaction [52].

### Ethics Approval and Consent to Participate

The study has been approved by the Swedish Ethical Review Authority (Dnr 2019-01320). All participants will give written informed consent prior to participation in any study procedure (focus group interview, heuristic evaluation, user test).

## Results

Recruitment started in May 2019. Data collection, analysis, and scientific reporting are expected to be completed in December 2019. The study was funded by a grant from the Swedish Research Council for Health, Working Life, and Welfare. Focus group interviews were being undertaken with high school students from October through December, and full results are expected to be published in Spring 2020. A planned clinical trial will commence in Summer 2020.

## Discussion

As a growing body of research suggests that health risk behaviors often do not occur in isolation, this study considers interventions that address lifestyle behaviors related to diet, physical activity, smoking, and alcohol. Also, more research is needed into the documentation and critical analysis of the formative research processes required in the development and refinement of effective mHealth interventions [24,26]. This protocol describes a participatory design study with the aim of developing an mHealth intervention to promote healthy lifestyles among high school students that can be delivered via school health staff. This protocol provides a scientific record of the methodologies used when developing the intervention in order to enhance transparency of research. Additionally, as described above, the LIFE4YOUth intervention program is part of a larger research program (funded by Forte, the Swedish funding agency for health and social affairs research) [28], and the formative work presented here will also be used as a framework for the other trials in the program.

Through formative research and participatory design, we believe this study will result in deepened knowledge regarding what aspects of content and structure end users (eg, high school students) consider important for designing mHealth lifestyle behavior interventions. More specifically, the study is expected to give answers as to whether an mHealth intervention that gives access to interactive and personal modules contained within a mobile phone–based dashboard is useful and accepted among high school students. This knowledge is valuable in order to guide further development of a final version of the novel mHealth intervention program LIFE4YOUth targeting high school students. An RCT will be conducted to determine the efficacy of the intervention. If found effective in the RCT, the program has the potential to be implemented nationally through school health services.

## Appendix

Multimedia Appendix 1 Interview guide.

## Abbreviations

COREQ: consolidated criteria for reporting qualitative research
mHealth: mobile health
NCD: noncommunicable disease
RCT: randomized controlled trial
SUS: system usability scale
WHO: World Health Organization
